# Leading from the Middle: Replication of a Re-Engagement Program for Veterans with Mental Disorders Lost to Follow-Up Care

**DOI:** 10.1155/2012/325249

**Published:** 2012-09-25

**Authors:** David E. Goodrich, Nicholas W. Bowersox, Kristen M. Abraham, Jeffrey P. Burk, Stephanie Visnic, Zongshan Lai, Amy M. Kilbourne

**Affiliations:** ^1^VA National Serious Mental Illness Treatment Resource and Evaluation Center and VA Center for Clinical Management Research, VA Ann Arbor Healthcare System, 2215 Fuller Road, Mailstop 152, Ann Arbor, MI 48105, USA; ^2^Department of Psychiatry, University of Michigan Medical School, North Campus Research Complex, 2800 Plymouth Road, Building 14, Ann Arbor, MI 48109-2800, USA; ^3^Mental Health Services, Patient Care Services, Veterans Health Administration, Washington, DC 20420, USA

## Abstract

*Objectives*. Persons with mental disorders experience functional impairments and premature mortality. Limited continuity of care may contribute to disparities in this group. We describe the replication of an evidence-based outreach program (Re-Engage) to reconnect Veterans with mental disorders into care who have dropped out of services. *Methods*. Using the Enhanced Replicating Effective Programs framework, population-based registries were used to identify Veterans lost-to-care, and providers used this information to determine Veteran disposition and need for care. Providers recorded Veteran preferences, health status, and care utilization, and formative process data was collected to document implementation efforts. *Results*. Among Veterans who dropped out of care (*n* = 126), the mean age was 49 years, 10% were women, and 29% were African-American. Providers determined that 39% of Veterans identified for re-engagement were deceased, hospitalized, or ineligible for care. Of the remaining 68 Veterans, outreach efforts resulted in contact with 20, with 7 returning to care. Providers averaged 14.2 hours over 4 months conducting re-engagement services and reported that gaining facility leadership support and having service agreements for referrals were essential for program implementation. *Conclusions*. Population-level, panel management strategies to re-engage Veterans with mental disorders are potentially feasible if practices are identified to facilitate national rollout.

## 1. Introduction

Persons with mental disorders (e.g., bipolar disorder, schizophrenia, and recurrent major depressive disorder) experience a disproportionate burden of functional impairment, morbidity, and premature mortality from preventable causes, notably heart disease [1, 2]. Acute mental health symptoms can predispose this population to gaps in care as well as increase access barriers due to stigma, limited insurance, or difficulty navigating health care systems [3]. 

Evidence shows that even in integrated health care systems, continuity of care remains problematic. For example, one national study of patients diagnosed with mental disorders in the Department of Veterans Affairs (VA) health care system found that 21% had experienced a 12-month gap in contact with the health care system while 42% had a 12-month gap in mental health care [4]. Similarly, another study found that VA patients with schizophrenia who had little VA utilization in the prior year had a twofold increased risk for death relative to patients without schizophrenia [5].

A persistent barrier to improving access and continuity of care among patients with mental disorders has been the lack of a systematic process for identifying and engaging those who have dropped out of care and providing meaningful data to frontline providers on the patients who are most at risk of poor outcomes [6, 7]. Subsequently, the VA Office of the Medical Inspector (OMI) launched a national quality improvement (QI) initiative in 2007 to determine whether targeted outreach services using VA registry data could improve patient access to medical care and reduce premature mortality in Veterans with mental disorders who were lost to follow-up [8]. VA administrative databases were used to identify Veterans with chronic mental disorders and points of contact at each VA facility attempted to contact these Veterans and re-engage them in VA care services. Of the 3,306 Veterans contacted over a 7-month period, 72% returned to VA care, and those returning to care experienced a 6-fold reduction in mortality risk compared to those not returning to care [8].

Subsequently, VA leaders sought to rapidly translate this initiative into routine care. In preparation for implementing and sustaining this new program, VA mental health leaders sought to replicate the QI initiative based on input from frontline providers as well as from facility- and regional-level leaders. The aim of this paper is to describe the adaptation and findings from a five-site pilot study involving the replication of this QI initiative (“Re-Engage”). We also describe the underlying implementation framework used to adapt Re-Engage that emphasizes input from frontline providers rather than a top-down mandate approach.

## 2. Materials and Methods

This longitudinal study assessed the implementation of the Re-Engage initiative. Similar to the original QI initiative described elsewhere [8], Re-Engage involved the use of VA national administrative data and local providers to identify and contact Veterans with mental disorders who were lost to follow-up (i.e., had not received VA services for at least one year). However, Re-Engage was adapted based on input from frontline providers using an implementation framework, described below. Five VA facilities participated in this initial replication of Re-Engage. Primary outcomes included the clinical and health status of Veterans with mental disorders lost to follow-up as well as the number of Veterans re-engaged in VA services after the implementation. We also conducted a formative evaluation based on assessment of field notes and provider interviews to inform a larger national dissemination of Re-Engage. A VA medical center (VAMC) Institutional Review Board evaluated the protocol for this evaluation and determined that it was a quality improvement effort and not a research study requiring informed consent. This project was covered by a global IRB to conduct *program *evaluations and secondary data analyses of VA mental health treatment programs. Hence, participating providers and VA leaders did not sign a consent form because the focus the study was on eliciting process data on the implementation of a VA clinical *program *as opposed recruiting providers to a research intervention in which VA providers or patients would be the focus of program outcomes. All patient-level outcomes were obtained as secondary data to the implementation of this clinical program.

### 2.1. Re-Engage Implementation Framework

**Figure 1:**
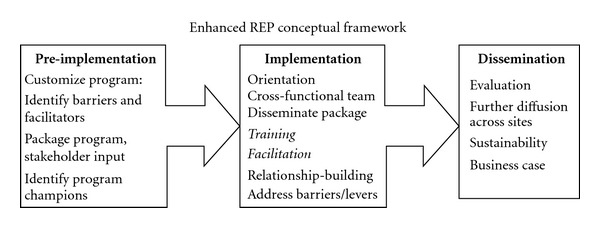


Re-Engage was implemented using the Enhanced Replicating Effective Programs framework (Enhanced REP) [9–11] (Figure 1). Enhanced REP is based on the Centers for Disease Control's (CDC) Research to Practice model [9, 11, 12] which incorporates principles from diffusion of innovations and social learning theories [13, 14]. REP was enhanced to address complex healthcare organizational relationships based on principles of Participatory Management [11, 15–19]. Specifically, Enhanced REP takes into account organizational barriers and facilitators to adoption of evidence-based practices through a combination of front-end training and technical assistance support and ongoing input and relationship building among frontline providers to promote effective practice. In this study, Enhanced REP was applied to implement Re-Engage prior to the national rollout of the program in order to identify barriers to implementation and to obtain input from provider endusers of the program.

Enhanced REP consists of three primary phases: (1) translation of program components into nontechnical, user-friendly language referred to as *packaging*, (2) *training* program adopters on how to implement the program and incorporating frontline input on its adaptation, and (3) supporting the *dissemination* of the program across multiple contexts through facilitation (Table 1). Enhanced REP facilitation [11] is an interactive process throughout all three phases that involves helping frontline providers build relationships with other existing providers and identifying opportunities to publicize or leverage the program's successes in order to increase leadership commitment and ultimately, adoption [20, 21]. Staff from the VA Serious Mental Illness Treatment Resource and Evaluation Center (SMITREC) acted as facilitators and provided guidance on implementing Re-Engage to the frontline providers between May and August 2011. Facilitators specifically provided the frontline providers guidance in: (1) identification and engagement of key stakeholders at all organizational levels, (2) identification of barriers to implementation of Re-Engage and strategies to overcome barriers, and (3) ongoing evaluation and adaptation of the implementation process to further enhance uptake (Table 1). Additionally, formative processes of program evaluation were built into Enhanced REP to help all stakeholders systematically monitor and track re-engagement efforts, identify barriers to program implementation so that alternative implementation strategies could be tested, and share successes with other sites and personnel [22, 23].

### 2.2. Setting and Procedures

Prior to initial replication of Re-Engage at each of the five sites, VA leaders applied Enhanced REP components to encourage input and engagement from frontline providers. They also identified local recovery coordinators (LRCs) as the natural points-of-contact for the Re-Engage initiative. LRCs are typically psychologists or social workers who facilitate the adoption of recovery-oriented services such as social skills training, psychoeducation, or peer support for patients with mental disorders at each VA facility [24]. LRC positions were mandated in VA in 2006 and were reaffirmed by the VA Uniform Mental Health Services Handbook [25] in 2008. VA Central Office leaders saw the Re-Engage initiative as an opportunity to help raise the visibility and clinical role of this essential VA mental health position.

This paper reports on the pilot implementation of the Re-Engage program by the LRCs at five VAMCs between May and August, 2011. Sites were identified by VA leaders based on their geographic representation that included small- to moderate-sized VA medical centers located in the southeastern and Midwestern United States. In addition, sites needed to have at least 20–30 patients meeting the criteria for program inclusion described below. Site-level characteristics are described in Table 2. A formal email invitation to the LRC and their respective regional mental health leader was sent by VA leadership. Specifically, three regional mental health leaders helped facilitate the recruitment of five LRCs at each of the five facilities. VA central office leaders included three senior leaders in VA mental health recovery services and five staff from VA SMITREC.

Consistent with Enhanced REP, staff from the VA SMITREC developed an initial implementation manual (package) detailing the Re-Engage program (Table 1). Beginning in March 2011, regular conference calls between national and local stakeholders occurred every 4–6 weeks to plan and implement Re-Engage. Provider training in the program was initiated in May 2011, starting with two 1.5-hour conference calls for the LRCs at the five demonstration sites. In addition, a 2-hour orientation and training presentation on Re-Engage was provided at a national LRC meeting in late June 2011 for all LRCs nationally, including the five LRCs who participated in this pilot demonstration program. Ongoing facilitation was provided via monthly conference calls from May through August 2011 and addressed provider and facility engagement (Table 1).

### 2.3. Participants

Veterans with mental disorders who were using VA services at one of the five sites, who had dropped out of care between FY 07 and FY 09, and who were still alive as of April 2011 based on VA Beneficiary Identification Locator System and Social Security Administration death files were identified by SMITREC staff using the VA National Patient Care Database [8]. Veterans with mental disorders included those with a diagnosis of bipolar disorder (International Classification of Diseases, Ninth Revision, Clinical Modification (ICD-9-CM) codes 296.0–296.8) or schizophrenia (ICD-9-CM codes 295.0–295.4; 295.6–295.9). Bipolar disorder and schizophrenia were the focus of the initial Re-Engage implementation due to the clinical severity of these diagnoses. As with the previous QI initiative [8], Veterans were considered to have dropped out of care if they had no VA contact for a minimum of one year and had no outpatient visits or an inpatient stay of two days or fewer within the VA health care system starting in May 2007 (end of the original QI project) through the end of FY 09 (September 30, 2009).

Lists of Veterans who had dropped out of care were created for each facility, and Veterans were assigned to the VA facility where they last received care. To prioritize re-engagement services for the pilot and national implementation, the lists focused on a subgroup of Veterans who dropped out of care and also had at least one inpatient hospitalization prior to drop out and who were less than 65 years of age (i.e., less likely to be in nursing home or covered by Medicare services). SMITREC staff generated and sent contact lists to the LRCs in May 2011. Veteran lists were merged with contact information ascertained from the VA National Enrollment Data file and sent through encrypted email to the five participating LRCs. A total of 126 Veterans were identified from the five pilot sites (range: 17–30 per site).

### 2.4. Implementation of Re-Engage

The five LRCs (one from each site) reviewed their lists and updated the Veterans' status using a secure web-based registry. The registry was housed on a VA intranet website that was developed using Inquiste Survey System TM 9.5 software. LRCs were instructed to update Veteran status using information available from local VA medical records and internet websites (e.g., local jail/state prison websites). Veterans found to be deceased, lacking contact information, ineligible for VA services, or receiving care through non-VA institutions were not contacted by the LRCs, but LRCs recorded the status of these Veterans using the web-based registry.

The LRCs contacted the remaining eligible Veterans by phone or mail to determine each Veteran's interest and/or need for continued VA treatment services. For all Veterans desiring VA care, the LRCs were asked to facilitate referrals to appropriate services and to help ensure an appointment was scheduled for the Veteran. All completed re-engagement efforts were documented in the web-based registry that enabled SMITREC staff to generate periodic feedback reports to all stakeholders.

### 2.5. Data Collection and Measures

 Quantitative data evaluating the replication of Re-Engage were ascertained from the web-based registry (patient outcomes). Formative evaluation data were also ascertained from interviews and field notes from the LRCs and program leadership.

#### 2.5.1. Quantitative Data

The LRCs documented all re-engagement services for each Veteran in the registry using a two-part reporting form. The first part included five structured questions that documented the LRCs' efforts in updating the Veteran's status. For Veterans alive and eligible for re-engagement attempts, the second part of the registry included questions that documented outreach attempts, whether contact was made, reasons for not contacting the Veteran, health status, and preference for health care services, whether the Veteran was referred for care, and reasons the Veteran did not want care. The LRCs were also asked to estimate the time expended implementing the Re-Engage program, conducting re-engagement efforts with Veterans, and documenting their efforts for workload capture purposes.

#### 2.5.2. Formative Evaluation

Detailed field notes and telephone call minutes were collected over time to record activities related to the application of the Enhanced REP framework, including collaboration between central and local program stakeholders regarding key implementation decisions, the facilitation process, feedback from stakeholders, and recommendations for protocol improvement to inform national implementation efforts. Beginning at pre-implementation of the pilot, SMITREC staff recorded all conference call meeting minutes that were verified by call participants to ensure accuracy. In addition, participatory feedback on the Re-Engage program was also garnered at the 2011 national LRC conference by having these providers meet in breakout sessions in which the program was presented, audience feedback was solicited, and concerns and suggestions were recorded on large flipchart pages to verify accuracy of provider comments. All technical assistance and facilitation interactions (predominantly emails) between SMITREC and local providers were treated as formative process data to refine outreach protocols.

### 2.6. Analyses

Descriptive statistics and bivariate analyses were computed for Veteran characteristics and provider (i.e., LRC) workload. All statistical analyses were performed using SAS 9.2 (SAS Institute, Cary, NC, USA). All formative processes data were analyzed inductively on an interactive basis to identify key issues and implementation themes using principles of grounded theory [26]. All process data were coded by two raters (investigators DEG, NWB) to identify key themes, and a third rater (AMK) helped achieve consensus in cases of disagreement. A fourth member of the research team (JPB) performed a member check to verify the validity of the qualitative themes for protocol improvement. Both process and quantitative data were then shared with participating local stakeholders for review and to verify the applicability of implementation protocols to the context of frontline providers.

## 3. Results

### 3.1. SMI Re-Engage Pilot Outreach Sample Characteristics

Veterans who initially dropped out of care and were identified for re-engagement (*N* = 126) were predominantly male (90%), had a mean age of 49 years (SD = 11), were Caucasian (71.4%), and unmarried (75.4%), reflecting similar demographic characteristics of all VA patients with SMI [27]. Fifty-two percent were diagnosed with bipolar disorder, and 66.7% were prescribed antipsychotic medications prior to becoming lost to follow up. Nearly a third had been hospitalized 2 or more times during their last year of VA care, with the last VA utilization being for inpatient treatment (32%). The majority (87.3%) had one or more diagnosed medical conditions, while 31.0% had service-connected disability of 70% or greater. Finally, 26.2% had used VA homelessness services in the years prior to their loss to care. Compared to the national cohort of Veterans with SMI (*n* = 241,976) [27], those who dropped out of care were more likely African-American (29% versus 22%), diagnosed with schizophrenia (48% versus 37%), or have prior history of homelessness (26% versus 13%).

### 3.2. Results of SMI Re-Engage Pilot Outreach Efforts

For outreach and re-engagement, 14 individuals were excluded because no contact information was available in national or local administrative databases (Table 3). Of the remaining 112 Veterans, 27 (24.1%) were determined to no longer be alive at the time of the outreach. An additional 11 Veterans (9.9%) were hospitalized (*N* = 6 (5.4%) in prison; *N* = 2 (1.8%) in non-VA hospital; *N* = 2 (1.8%) in nursing home; *N* = 1 (0.9%) in VA hospital), and 4 (3.6%) were no longer eligible for VA care. Two Veterans (1.8%) re-engaged in VA care independently prior to the outreach. A total of 68 Veterans (60.7%) remained within the sample targeted for re-engagement.

The 68 Veterans were initially contacted by telephone (*N* = 67, 98.5% of sample) and then mailed letters (*N* = 51, 75.0% of sample). The LRCs also attempted to make contact through the use of Internet searches (*N* = 13, 19.1% of sample), by contacting next of kin (*N* = 13, 18.8% of sample), or by contacting Veterans' spouses (*N* = 2, 3.4% of sample).

Through outreach efforts, the LRCs were able to establish contact with 20 (34.5%) of these 68 Veterans. Among the remaining 48 Veterans, 17 (35.4% of those not contacted) were no longer available at their listed phone number/address, 23 (47.9%) had inaccurate contact information, 6 (12.5%) were unresponsive to being contacted, and 2 (4.2%) were not contacted as they received VA care independently during the outreach process.

Veterans contacted through outreach efforts indicated a variety of service needs. Seven Veterans (35.0%) reported a mental health service need, 7 (35.0%) reported a need for medical care, 5 (25.0%) required assistance with employment, 3 (15.0%) asked for assistance with transportation, and 1 (5.0%) requested legal assistance. Four Veterans (20.0%) did not require any VA services at the time of outreach. At the time of outreach, 7 Veterans (35.0%) reported taking an appropriate medication regimen while an additional 7 (35.0%) were taking no psychotropic medications. Six Veterans (30.0%) did not discuss their medication arrangement during the outreach process. One Veteran (5.0%) was at-risk for becoming homeless at the time of the outreach.

The majority of the 20 contacted Veterans rated their physical health as *fair* (*N* = 10, 50.0%), with an additional 4 (20.0%) in *good* health, 3 (15.0%) in *very good* health, and 3 (15.0%) in *poor* physical health. Similarly, the majority (*N* = 12, 60.0%) rated their mental health as “*fair*,” with 3 (15.0%) “*good*,” 2 (10.0%) “*very good*,” 1 (5.0%) “*excellent*,” and 2 (10.0%) “*poor*” in terms of mental health functioning.

After establishing contact, the LRCs attempted to schedule appointments with VA providers for Veterans contacted during the outreach process. Of the 20 Veterans contacted, appointments were scheduled for 5 (25.0% of those contacted during outreach), with 15 (75.0%) unable to be matched to VA appointment. Of the 5 Veterans connected to VA appointments, 3 (60.0%) were scheduled a primary care appointment, 3 (60.0%) were scheduled a mental health appointment, and 1 (20.0%) was scheduled an appointment with VA housing services.

The LRCs indicated multiple reasons for a lack of appointment following outreach contact based on Veteran responses. Appointments were not scheduled for 5 Veterans (33.3% of those contacted but not scheduled) due to a lack of actual or perceived need for VA services by the Veteran. Four Veterans (26.7%) reported a preference for non-VA care. Two Veterans (13.3%) indicated a preference for a walk-in rather than scheduled care. Three Veterans (20.0%) were located in a treatment area outside of the VA facility's catchment area and were given information allowing them to reestablish care at the treatment center nearer to them. One Veteran indicated that distance/transportation presented a barrier that would prevent them from participating in VA care at this time.

### 3.3. Workload Associated with Outreach Efforts

Across the five pilot sites, the LRCs reported an average of 5.1 hours (range: 1.5–3.0 hours) spent setting up outreach efforts, 10.6 hours (range: 6.7–16.0 hours) spent engaging in outreach efforts, and 1.3 hours (range 0.5–2.0 hours) spent documenting outreach efforts. Overall, sites reported that outreach efforts required an average of 14.2 hours, with a range of 8.2 to 19.0 hours across the sites participation in the re-engagement outreach efforts.

### 3.4. Formative Evaluation

LRCs identified three core strategies to facilitate re-engaging Veterans with mental disorders: (1) utilize internal sources of data to locate updated contact information; (2) utilize external data sources to locate Veterans; (3) carefully track outreach efforts (Table 4).

LRCs also identified several barriers to Veteran appointment attendance following successful outreach contact. Four central barriers were identified: (1) reluctance to prioritize treatment slots for these Veterans; (2) problems achieving timely follow-up appointments with chronically backlogged services; (3) transportation issues experienced by Veterans living in rural areas; and (4) difficulties coordinating appointments between multiple sites/treatment teams (Table 5). However, the LRCs and facilitators also identified a number of strategies for overcoming these barriers (Table 5).

Overall, among Veterans who desired to be seen, local providers faced barriers to making appointments primarily because they did not have control over the appointment scheduler and because of limited clinic slots. Nonetheless, key strategies identified by local providers that facilitated appointment scheduling included leveraging relationships with other coordinators who were part of VA programs that needed to demonstrate workload, including mental health care management (Primary Care-Mental Health Integration) [28] and homelessness programs.

## 4. Discussion

To our knowledge, Re-Engage is the first example of a national population management program that has been implemented to improve care for persons with mental disorders. The use of formative pilot work and local provider engagement was essential to translating this promising quality improvement research into clinical practice and ensuring its successful replication over time. This study reports on the initial replication of this program in order to inform the national rollout of Re-Engage, which was approved as a national policy directive in January 2012 by VA Central Office. The Enhanced REP implementation framework provided a framework with which to develop the Re-Engage handbook, training program, and facilitation program, as well as garner feedback from providers and leaders throughout the implementation process. Notably, facilitation provided by SMITREC staff and VA leaders encouraged local providers (LRCs) to engage with their local leaders and other local providers to enhance opportunities to identify and bring back into care Veterans who had been lost to followup.

To date, this is one of the first studies to implement a population management model (i.e., use of electronic registries to identify and intervene on high-risk patients) to promote outreach and re-engagement in care for persons with mental disorders. Previous efforts to implement quality improvement initiatives focusing on services for persons with mental disorders have mainly focused on preventing rehospitalizations (e.g., [29, 30]). We applied, a theory-based implementation framework that included guidance on generating program buy-in at the local level (facilitation). SMITREC staff members' role as facilitators allowed for the cross-site identification of barriers and potential solutions as well as feedback from local sites on successful practices, and this formative information was subsequently used to inform the process of national program implementation.

In comparison with the original QI project previously implemented by the original VA OMI initiative [8], this pilot project had a lower percentage of Veterans re-engaged in care (10% versus 72%, resp.), even though initial rates of attempted contacts were similar (61% versus 68%). These differences point to a number of challenges when replicating an established program once the mandate for the original quality improvement effort had ended. First, unlike the OMI project which implemented outreach to all Veterans with schizophrenia and bipolar disorder who were lost to care, this pilot project targeted Veterans who were believed to be at greatest risk for poor outcomes (prior hospitalization history), and hence, a greater percentage of Veterans in this pilot may have been more difficult to contact and re-engage. In addition, the replication study had a shorter follow-up period (the LRCs were given less than 3 months to contact versus 7 months in the original QI initiative). Moreover, the original OMI initiative was implemented when the both LRC positions were relatively new, and the VA had recently expanded mental health hiring. Hence, time commitments and priorities probably enabled full attention to the quality improvement initiative at the time of its initial implementation.

Through the process of replicating Re-Engage, we learned several lessons that will be applicable not only to the national implementation of Re-Engage but to the implementation of other large-scale, population-based outreach programs. First, top-down support for initiatives such as Re-Engage is crucial, but frontline support is also needed to maintain enthusiasm and provider buy-in over time. At the time of the pilot, the Directive for the Re-Engage project had not undergone final concurrence (approval) for national implementation; hence, some LRCs may have met resistance in obtaining appointment times for Veterans lost to follow-up due to competing priorities. Nonetheless, the LRCs were able to identify workarounds to leverage appointments with existing priorities such as the VHA's mandate to see Veterans with a mental health need within 14 days. This ability to “lead from the middle,” while observed in other implementation efforts [31, 32], is a potentially complementary factor to top-down mandates to enhance the uptake of quality improvement initiatives.

In addition, local provider input was essential in creating practical documentation tools that were necessary for the program's eventual national rollout, including a web-based survey that can adequately capture the complexity of Veteran re-engagement cases and simplify outreach documentation. The data gathered through local providers' outreach efforts were also used to improve the accuracy of Veterans' current information and status in VA administrative databases. Moreover, through local providers' documentation, we were able to capture the amount of time they allocated to the program. Providers' ability to complete the program tasks in a relatively small amount of time suggests that the processes piloted may help to increase the sustainability of Re-Engage. Additional improvements to Re-Engage include the simplification of the web-based registry tool, establishment of national benchmarks for Re-Engage uptake (including percentage of Veterans with updated information on status, percent in which contact was attempted, and percent referred to for care), and the development of an LRC mentoring team. These features are currently being tested in the national rollout of Re-Engage.

Although the present study yields useful information regarding how to pragmatically and rapidly implement a new national policy at local sites, there are several limitations that warrant acknowledgement. Given the pilot nature of the project, the sample of Veterans identified for outreach was small, and only five sites participated in the pilot. Although this may limit the generalizability of results, it is also important to acknowledge that the experiences of the local providers at these five sites yielded important formative information which will be used to improve the larger implementation process of Re-Engage. Similarly, the practical implementation of Re-Engage lacked the rigorous design and data collection methods that characterize randomized controlled trials, making it difficult to infer the causal influence of the intervention. In addition, the findings from this implementation study may not generalize to settings outside of a closed healthcare system such as the VA, which has a common electronic medical record system and access to a national provider network. Despite these limitations, findings from this study will likely enhance the translation of Re-Engage into practice and inform additional efforts to establish implementation strategies at the national level.

## 5. Conclusions

On January 10, 2012, VHA Directive 2012-002 was signed by the VA Undersecretary for Health authorizing Re-Engage to become standard clinical practice to promote continuity of care among Veterans diagnosed with mental disorders. While the goal is to first contact Veterans with major mental disorders including bipolar disorder, VA leaders have discussed the expansion of Re-Engage to other Veterans with major depression, PTSD, or other mental disorder diagnoses, once the national rollout has been fully implemented. These initiatives are crucial steps toward the promotion of measurement-based care and ultimately, improved quality and outcomes for persons with mental disorders [6, 7]. Overall, this implementation pilot demonstrated the potential barriers to implementing a national initiative when no national mandate exists, as well as solutions that involve facilitating partnerships at the local level to empower frontline providers. Moreover, relatively straightforward information technology resources such as administrative databases and web-based surveys can be used to implement population management in mental healthcare settings, thus encouraging this cultural shift towards systems redesign. Further support in building relationships with other providers to effectively refer Veterans to care in a timely manner (i.e., “leading from the middle”) is needed to sustain the program and subsequent systems redesign. Ongoing evaluation of the national implementation and outcomes of Re-Engage will ultimately determine the most successful strategies for using population-based registries to improve health outcomes for persons with mental disorders.

## Figures and Tables

**Table 1:** Application of the Enhanced REP framework to Re-Engage.

Enhanced REP component	Key processes	Re-Engage activities
Preimplementation		

Customize the evidence-based practice	Conduct organizational needs assessment of key personnel Working with facilitators, collaborate to make site-specific intervention adaptations (i) Organizational structure (ii) Impetus to transform (iii) Perceptions of the identified problem (iv) Site-specific adaptations Creation of packaged, user-friendly implementation manual/tool kit	Reviewed VA Office of the Medical Inspector QI Project report findings Created demonstration collaborative to pilot Re-Engage protocols (i) “Lead from middle” model (ii) Draft of VA program directive and operational plan (iii) Formative assessment of pilot stakeholders' perceptions (iv) Specified core versus modifiable elements Revised, expanded, and enhanced QI implementation manual/form

Identify champions	Facilitators work with national and local leaders to identify early adopters, past performance	Five LRCs and sites identified based on leader input

Implementation		

Training	(i) Facilitators and VA leaders provide targeted presentations to key leaders and early adopters (ii) Facilitators provide site-specific and staff level appropriate customized training	(i) Conference calls with national, regional, and facility-level leaders (ii) Lead program overview to local recovery coordinators and local leaders at conferences or on conference calls

Orientation	Facilitators organize resources to support implementation with provider input: (i) Staff handbook (ii) Service agreements (iii) Service line leaders Advertise and publicize the program local stakeholders (i) Make an empirical case for program (administrators, staff) (ii) Highlight the potential benefits of the program to the Veterans, VAMC, and VSOs (iii) Advertise (newsletters, poster boards, etc.)	Created Re-Engage handbook with implementation checklists to promote interdisciplinary coordination: (i) referral scheduling, service agreements, and service line leaders/facility directors Developed advertising resources that enabled the LRCs to “lead from the middle” and encourage support for Re-Engage through the following means: (i) Present data from original study project regarding mortality reductions (ii) Educate clinic personnel of program benefits-information sheet (iii) Make in-service presentations—talking points sheet (iv) Share flyers with community partners and patients

Facilitation	Start program Utilize local resources and sustain advertising. Facilitators continue to provide technical assistance to frontline providers to problem solve implementation issues Collect data to measure program impact and to enhance program delivery	(i) Provide adaptable marketing tools and coordinate with services with similar goals (e.g., homelessness) (ii) Facilitators hold regular calls with frontline LRCs (iii) Continuous data collection and implementation of monthly reports and online feedback interface for local providers and policy leaders

Evaluation and sustainability	Reevaluate program successes and ways the program could be further adapted to improve outcomes and customer satisfaction at the site.	Pilot findings reviewed with demonstration sites and national LRC network to: (i) Enhance implementation guide and advertising resources (ii) Provide examples of best practices of local communication and coordination (iii) Revised assessment form and feedback tool (iv) Review business case for ongoing programming

**Table 2:** Site-level characteristics of VA healthcare systems participating in piloting Re-Engage.

Site	Unique patients	Number of CBOCs^a^	Number of inpatient Beds^b^	Recovery center present^c^	LRC professional background
1	37,018	2	0	No	LCSW
2	56,465	3	105	Soon	Psychologist
3	37,658	2	315/155	Soon	LCSW
4	48,767	3	271	Yes	Psychologist
5	50,730	9	192	Yes	LCSW

^a^CBOC: community-based outpatient clinic.

^b^Includes medical and psychiatric acute care beds.

^c^Recovery center—Presence or planned implementation of a psychosocial rehabilitation and recovery center for veterans that are mandated services for VA medical centers serving a large number of veterans with SMI (e.g., greater than 1,000 unique patients per year).

**Table 3:** Veteran status determined through re-engagement contacts (*N* = 112).

Veteran status	*N*	%
Inappropriate for re-engagement efforts	44	39.3
Deceased at time of re-engagement	27	24.1
Incarcerated in jail or prison	6	5.4
Hospitalized or housed in institution	5	4.4
Ineligible for VHA services	4	3.6
Veteran re-engaged in VA care independently	2	1.8
Appropriate for re-engagement services	68	
Veteran unsuccessfully contacted	48	70.6
Veteran contacted by phone, mail, or other modality	20	29.4
Services requested by veterans at time of re-engagement contact^a^	20	
Mental health	7	35.0
Medical care	7	35.0
Employment assistance	5	25.0
Transportation	3	15.0
Daily needs (e.g., food, clothing, housing)	3	15.0
Legal services	1	5.0
No services requested at time of re-engagement contact	4	20.0
Result of re-engagement contact	20	
Appointment scheduled	5	25.0
Veteran declined to schedule appointment at time of contact	15	75.0

^a^Percentages do not total 100% as most veterans indicated multiple areas of need.

**Table 4:** Recommended strategies to contact Veterans or verify Veteran status.

Utilize internal sources of data to locate updated contact information	
(1) Existing notes related to social work interventions often contain current contact information	
(2) Psychological assessments regularly contain updated patient contact information	
(3) Most recent discharge planning may contain current contact information	
(4) Recent treatment notes often contain current contact information that has not been updated in patients' overall information	
Utilize external data sources to locate patients	
(1) Review local newspaper databases for patient information (e.g., obituaries, marriages notices, etc.) that are not always reflected in patient charts and cross reference with telephone books	
(2) Access state and local websites for the status of incarcerated veterans	
(3) Telephone-based information services (e.g., 411) can provide patients' last known phone number	
Carefully track efforts aimed at contacting patients	
(1) Maintain a running log of attempts to re-engage patients, including dates and methods of outreach	
(2) Utilize certified mail as a way to verify if the patient received the letter (and verification of address)	

**Table 5:** Barriers and solutions related to appointment attendance following outreach contact.

Barriers	Solutions
(1) Service chiefs are reluctant to prioritize spots to re-engagement patients	(1) Emphasize incentives for timely appointments

(2) Difficult to achieve timely referral appointments for chronically backlogged services	(2a) Coordinate referrals through integrated care teams to increase ability to deliver immediate care
	(2b) Set up appointments between patient and outreach provider as a last resort

(3) Patients have difficulty attending appointments due to transportation issues (e.g., rural settings)	(3a) Proactively identify and coordinate resource to address logistical barriers (e.g., transports) to support referral uptake
	(3b) Outreach staff work with patients to identify and problem-solve logistical issues related to appointment attendance

(4) Coordinating referrals, appointments, and follow up with distant facilities can be challenging	(4a) Establish within network referral protocols and network with other points of contact to facilitate patient re-engagement
	(4b) Re-engagement staff directly facilitate the scheduling of appointments between patients and needed clinics
	(4c) Clearly document appointments and referrals within VA electronic medical record

## References

[1] Kilbourne AM, Morden NE, Austin K (2009). Excess heart-disease-related mortality in a national study of patients with mental disorders: identifying modifiable risk factors. *General Hospital Psychiatry*.

[2] Saha S, Chant D, McGrath J (2007). A systematic review of mortality in schizophrenia: is the differential mortality gap worsening over time?. *Archives of General Psychiatry*.

[3] Horvitz-Lennon M, Kilbourne AM, Pincus HA (2006). From silos to bridges: meeting the general health care needs of adults with severe mental illnesses. *Health Affairs*.

[4] McCarthy JF, Blow FC, Valenstein M (2007). Veterans affairs health system and mental health treatment retention among patients with serious mental illness: evaluating accessibility and availability barriers. *Health Services Research*.

[5] Copeland LA, Zeber JE, Rosenheck RA, Miller AL (2006). Unforeseen inpatient mortality among veterans with schizophrenia. *Medical Care*.

[6] Harding KJ, Rush AJ, Arbuckle M, Trivedi MH, Pincus HA (2011). Measurement-based care in psychiatric practice: a policy framework for implementation. *Journal of Clinical Psychiatry*.

[7] Pincus HA, Spaeth-Rublee B, Watkins KE (2011). Analysis and commentary: the case for measuring quality in mental health and substance abuse care. *Health Affairs*.

[8] Davis CL, Kilbourne AM, Pierce JR (2012). Reduced mortality among VA patients with schizophrenia or bipolar disorder lost to follow-up and engaged in active outreach to return to care. *American Journal of Public Health*.

[9] Kilbourne AM, Neumann MS, Pincus HA, Bauer MS, Stall R (2007). Implementing evidence-based interventions in health care: application of the replicating effective programs framework. *Implementation Science*.

[10] Kilbourne AM, Goodrich DE, Clogston J Randomized controlled trial of the Replicating Effective Programs implementation framework: the Recovery-Oriented Collaborative Care Study.

[11] Kilbourne AM, Neumann MS, Waxmonsky J (2012). Public-academic partnerships: evidence-based implementation: the role of sustained community-based practice and research partnerships. *Psychiatric Services*.

[12] Neumann MS, Sogolow ED (2000). Replicating effective programs: HIV/AIDS prevention technology transfer. *AIDS Education and Prevention*.

[13] Rogers E (2003). *Diffusion of Innovations*.

[14] Bandura A (1977). Self-efficacy: toward a unifying theory of behavioral change. *Psychological Review*.

[15] Valentine NM (1996). A national model for participative management and policy development. *Nursing administration quarterly*.

[16] Leana CR, Florkowski : GW (1992). Employee involvement programs: integrating psychological theory and management practice. *Research in Personnel and Human Resources Management*.

[17] Roethlisberger F, Dickson W (1939). *Management and the Worker*.

[18] Locke E, Schweiger D (1979). Participation in decision making: one more look. *Research in Organizational Behavior*.

[19] Lindenfeld S, Vlchek D (2001). Engaging physicians in continuous quality improvement. *Advances in Renal Replacement Therapy*.

[20] Stetler CB, Damschroder LJ, Helfrich CD, Hagedorn : HJ (2011). A Guide for applying a revised version of the PARIHS framework for implementation. *Implementation Science*.

[21] Stetler CB, Mittman BS, Francis J (2008). Overview of the VA quality enhancement research initiative (QUERI) and QUERI theme articles: QUERI series. *Implementation Science*.

[22] Gilbody S, Bower P, Fletcher J, Richards D, Sutton AJ (2006). Collaborative care for depression: a cumulative meta-analysis and review of longer-term outcomes. *Archives of Internal Medicine*.

[23] Stetler CB, Legro MW, Wallace CM (2006). The role of formative evaluation in implementation research and the QUERI experience. *Journal of General Internal Medicine*.

[24] Goldberg RW, Resnick SG (2010). US Department of Veterans Affairs (VA) efforts to promote psychosocial rehabilitation and recovery. *Psychiatric Rehabilitation Journal*.

[25] http://www.va.gov/vhapublications/ViewPublication.asp?pub_ID=1762.

[26] Forman J, Damschroder LJ, Jacoby L, Siminoff L (2008). Qualitative content analysis. *Empirical Research For Bioethics: A Primer*.

[27] Blow FC, Mccarthy JF, Valenstein M, Bowersox NW, Visnic : S Care for veterans with psychosis in the veterans health administration, FY10.

[28] Zeiss AM, Karlin BE (2008). Integrating mental health and primary care services in the department of veterans affairs health care system. *Journal of Clinical Psychology in Medical Settings*.

[29] Hwang SW, Weaver J, Aubry T, Hoch JS (2011). Hospital costs and length of stay among homeless patients admitted to medical, surgical, and psychiatric services. *Medical Care*.

[30] Gilmer TP, Manning WG, Ettner SL (2009). A cost analysis of san diego county’s REACH program for homeless persons. *Psychiatric Services*.

[31] Parker LE, Kirchner JE, Bonner LM (2009). Creating a quality-improvement dialogue: utilizing knowledge from frontline staff, managers, and experts to foster health care quality improvement. *Qualitative Health Research*.

[32] Rubenstein LV, Parker LE, Meredith LS (2002). Understanding team-based quality improvement for depression in primary care. *Health Services Research*.

